# Germination Enhances Phytochemical Profiles of Perilla Seeds and Promotes Hair Growth via 5α-Reductase Inhibition and Growth Factor Pathways

**DOI:** 10.3390/biology14070889

**Published:** 2025-07-20

**Authors:** Anurak Muangsanguan, Warintorn Ruksiriwanich, Pichchapa Linsaenkart, Pipat Tangjaidee, Korawan Sringarm, Chaiwat Arjin, Pornchai Rachtanapun, Sarana Rose Sommano, Korawit Chaisu, Apinya Satsook, Juan Manuel Castagnini

**Affiliations:** 1 Department of Pharmaceutical Sciences, Faculty of Pharmacy, Chiang Mai University, Chiang Mai 50200, Thailand; anurak_m@cmu.ac.th (A.M.); pichchapa_li@cmu.ac.th (P.L.); 2 Cluster of Valorization and Bio-Green Transformation for Translation Research Innovation of Raw Materials and Products, Chiang Mai University, Chiang Mai 50200, Thailand; korawan.s@cmu.ac.th (K.S.); sarana.s@cmu.ac.th (S.R.S.); 3 Cluster of Agro Bio-Circular-Green Industry (Agro BCG), Chiang Mai University, Chiang Mai 50200, Thailand; pipat.t@cmu.ac.th (P.T.); pornchai.r@cmu.ac.th (P.R.); 4 Faculty of Agro-Industry, Chiang Mai University, Chiang Mai 50100, Thailand; korawit.chaisu@cmu.ac.th; 5 Department of Animal and Aquatic Sciences, Faculty of Agriculture, Chiang Mai University, Chiang Mai 50200, Thailand; chaiwat.arjin@cmu.ac.th; 6 Department of Plant and Soil Sciences, Faculty of Agriculture, Chiang Mai University, Chiang Mai 50200, Thailand; 7 Office of Research Administration, Chiang Mai University, Chiang Mai 50200, Thailand; apinya.satsook@cmu.ac.th; 8 Research Group in Innovative Technologies for Sustainable Food (ALISOST), Department of Preventive Medicine and Public Health, Food Science, Toxicology and Forensic Medicine, Faculty of Pharmacy, Universitat de València, Avenida Vicent Andrés Estellés s/n, 46100 Burjassot, Spain; juan.castagnini@uv.es

**Keywords:** androgenetic alopecia, hair growth promotion, perilla seed (*Perilla frutescens* (L.) *Britt*.), screw compression (SC), selenium, supercritical fluid extraction (SFE), Sonic Hedgehog, Wnt/β-catenin, 5α-reductase inhibition

## Abstract

Seed germination is recognized for enhancing the accumulation of bioactive compounds. *Perilla frutescens* (L.) Britt., commonly known as perilla seed, is rich in fatty acids that may be beneficial for anti-hair loss. This study investigated the hair regeneration potential of perilla seed extracts—non-germinated (NG-PS) and germinated in distilled water (0 ppm selenium; G0-PS), and germinated with 80 ppm selenium (G80-PS)—obtained from supercritical fluid extraction (SFE) and screw compression (SC). SFE extracts exhibited significantly higher levels of polyphenols, tocopherols, and fatty acids compared to SC extracts. Among the germinated groups, G0-PS showed the highest bioactive compound content and antioxidant capacity. Remarkably, treatment with SFE-G0-PS led to a significant increase in the proliferation and migration of hair follicle cells, reaching 147.21 ± 2.11% (*p* < 0.05), and resulted in complete wound closure. In addition, its antioxidant and anti-inflammatory properties were reflected by a marked scavenging effect on TBARS (59.62 ± 0.66% of control) and suppressed nitrite amounts (0.44 ± 0.01 µM). Moreover, SFE-G0-PS markedly suppressed *SRD5A1-3* gene expression—key regulators in androgenetic alopecia—in both DU-145 and HFDPCs, with approximately 2-fold and 1.5-fold greater inhibition compared to finasteride and minoxidil, respectively. Simultaneously, it upregulated the expression of hair growth-related genes, including *CTNNB1*, *SHH*, *SMO*, *GLI1*, and *VEGF*, by approximately 1.5-fold, demonstrating stronger activation than minoxidil. These findings suggest the potential of SFE-G0-PS as a natural therapeutic agent for promoting hair growth and preventing hair loss.

## Simple Summary

Hair loss is a common concern affecting individuals of all ages. This study investigated the potential of *Perilla frutescens* seed extracts as a natural approach to support hair growth. The seeds were found to be rich in beneficial compounds, including antioxidants and essential fatty acids. Extracts obtained through supercritical fluid extraction contained the highest levels of these compounds and demonstrated superior biological activity. Notably, the extract from seeds germinated in distilled water showed the most promising effects in in vitro tests. It enhanced the growth, migration, and survival of hair follicle cells, reduced oxidative stress and inflammation, and activated genes involved in hair regeneration. These findings highlight the importance of extraction and germination techniques in enhancing the efficacy of plant-based ingredients. The results support the potential application of perilla seed extract as a natural, effective candidate for preventing hair loss and promoting hair growth.

## 1. Introduction

Androgenetic alopecia (AGA), also known as pattern baldness, is the most common form of hair loss. It is characterized by progressive hair thinning and loss of terminal hair after maturity, affecting a large number of people worldwide, with an estimated prevalence of 30–50% in both males and females. Hair undergoes a cyclical process comprising four key stages: the anagen phase, during which active hair growth takes place; the catagen phase, representing a brief period of regression; the telogen phase, a resting stage; and the exogen phase, during which the hair is ultimately shed. In the case of AGA, androgen hormones, particularly dihydrotestosterone (DHT), play a dominant role. DHT is synthesized from testosterone through the action of 5α-reductase (SRD5A) enzymes. When DHT accumulates in hair follicle regions that are hormonally sensitive, it disrupts the anagen phase prematurely. As a result, hair follicles progressively shrink, making the hair progressively thinner and shorter until growth ceases entirely [1]. Moreover, oxidative stress has been implicated in accelerating this degenerative process. Elevated DHT levels may increase nitric oxide (NO) production in hair follicles, triggering inflammatory cascades that contribute to further follicular damage and ultimately worsen the severity of hair loss [2].

Indeed, the formation of hair follicles is regulated by the interactions between epithelial and mesenchymal cells, mediated by signaling pathways originating from human hair follicle dermal papilla cells (HFDPCs). Hair regeneration is relevant to the modulation of several signaling pathways, notably Wnt/β-catenin (CTNNB1), Sonic Hedgehog (SHH, SMO, and GLI1), and vascular endothelial growth factor (VEGF) [3]. Among these, the Wnt/β-catenin pathway is essential for initiating hair regrowth. It promotes the accumulation of β-catenin in the cytoplasm of hair follicle cells, which in turn stimulates their proliferation and migration, crucial processes necessary for new hair formation [1]. Moreover, Wnt/β-catenin signaling positively influences the Sonic Hedgehog cascade, which plays a critical role during the mid-growth phase by promoting the proliferation of various cell types, including epithelial, fibroblast, and mesenchymal cells [4]. In parallel, VEGF enhances nutrient and oxygen delivery to hair follicles by increasing blood supply during the active growth phase, thereby creating a favorable environment for hair growth [5].

Currently, various treatment options are available for AGA, including pharmaceutical therapies such as topical minoxidil and oral finasteride, along with non-pharmaceutical approaches including laser therapy, regenerative cell therapy, gas therapy, and microneedling. According to current treatment guidelines for AGA, pharmaceutical therapies remain the primary approach [6]. Topical minoxidil is widely employed in both men and women, primarily by indirectly stimulating angiogenesis via VEGF upregulation. Finasteride, an inhibitor of 5α-reductase type 2 (SRD5A2), is used for both benign prostatic hyperplasia and AGA by reducing DHT levels within the cytoplasm of HFDPCs [1]. Despite their efficacy, these medications are associated with adverse effects: topical agents may cause dermatological irritation, while oral administration of finasteride has been associated with systemic effects, including sexual dysfunction and gynecomastia. As a result, there is growing interest in plant-derived compounds with anti-hair loss and hair growth-promoting properties as natural product-based therapies with more favorable pharmacological and safety profiles for AGA treatment.

Among the several phytochemicals synthesized by plants are polyphenols, fatty acids, alkaloids, terpenoids, and tocopherols, all of which exhibit specific biochemical properties and play unique roles in biological systems. These properties are governed by their chemical structures, which influence their solubility, reactivity, and biological activity within cellular systems [7]. *Perilla frutescens* (L.) *Britt*., traditionally known as Zisu and used in Chinese medicine, belongs to the Lamiaceae family and is widely cultivated across East Asia [8]. The northern region of Thailand is the primary area for perilla cultivation. Previous research has shown that perilla seeds contain a significant amount of oil (30–45% of seed weight), which contains diverse bioactive compounds, including polyphenols with strong antioxidant activity, as well as alkaloids, steroids, quinones, and tocopherols [9]. In addition, perilla seed oil contains various fatty acids, with a particularly high proportion of polyunsaturated fatty acids (PUFAs), notably α-linolenic acid, which comprises over 50% of the total oil content, along with linoleic acid [10]. Previous research has also highlighted the potential of these bioactive compounds in hair regenerative properties through modulation of Wnt/β-catenin, Sonic Hedgehog, and angiogenesis pathways [11]. Notably, our previous report also revealed that perilla seed extract significantly downregulated *SRD5A* genes (types 1–3), indicating its potential in inhibiting DHT-related hair loss.

Germination is commonly used to produce edible seeds and sprouts for consumption. This process allows many seeds to sprout quickly, enhancing their nutritional and medicinal properties. The influence of selenium during seed germination has gained increasing attention due to its beneficial effects on plant development. Selenium application during germination enhances seed vigor and stress tolerance, promoting healthier growth and improved resilience to environmental stressors. These effects are primarily attributed to selenium’s ability to activate antioxidative defense mechanisms, mitigate oxidative damage—such as that caused by ultraviolet radiation—and delay senescence [12,13]. Previous studies have reported that germination with selenium enhances the phytochemical content in plant seeds and improves the antioxidant efficacy of seed-derived extracts [14,15]. In this study, perilla seeds were germinated for 7 days either in distilled water (0 ppm selenium; G0-PS) or in selenium solution at 80 ppm (G80-PS) compared with the non-germinated control (NG-PS). This approach aimed to enhance both the levels of bioactive compounds and the antioxidant capacity of *perilla* seeds.

At present, screw compression (SC) and supercritical fluid extraction (SFE) are two widely used methods for obtaining oil from plant seeds. SC is recognized as an effective mechanical technique, capable of recovering 75–90% of the oil content. It is often favored for its ability to preserve heat-sensitive bioactive compounds, including antioxidants, thereby enhancing the nutritional and functional qualities of the extracted oil. In contrast, SFE is an emerging green technology that offers several advantages over conventional extraction methods. Operating at relatively low temperatures and without the need for organic solvents, SPE produces high-purity extract free from solvent residues, making it particularly suitable for pharmaceutical and cosmetic applications. Moreover, as a food-grade, solvent-free method, SFE effectively preserves bioactive constituents by minimizing thermal and oxidative degradation during the extraction process [16,17]. Based on our previous findings, both SC and SFE outperformed conventional extraction in terms of yield, bioactive constituent retention, antioxidant capacity, and suppression of genes in the androgen pathway. Therefore, these two methods were selected for further investigation in the present study.

To better understand the factors affecting the phytochemical profile of perilla seeds, this study investigated the effects of germination and various extraction techniques on the accumulation of bioactive constituents. The obtained extracts were subsequently examined for their protective effects against oxidative damage and inflammatory responses, along with their ability to affect major signaling pathways associated with hair regrowth, namely Wnt/β-catenin, Sonic Hedgehog, and angiogenesis, while also suppressing hair loss stimulators, particularly the 5α-reductase (SRD5A) enzyme.

## 2. Materials and Methods

### 2.1. Chemicals and Reagents

A range of analytical reagents, including the Folin–Ciocalteu reagent, hydrogen peroxide (H_2_O_2_), trichloroacetic acid (TCA), and Triton X-100, were purchased from Merck (Darmstadt, Germany). Sigma Chemical (St. Louis, MO, USA) supplied the antioxidant and standard reagents, including epigallocatechin gallate, gallic acid, Trolox (6-hydroxy-2,5,7,8-tetramethylchroman-2-carboxylic acid), 2,2′-azino-bis (ethylbenzthiazoline-6-sulfonic acid) (ABTS), 2,2-diphenyl-1-picrylhydrazyl (DPPH), sulforhodamine B (SRB), diclofenac sodium, L-ascorbic acid, and lipopolysaccharide (LPS). Thiobarbituric acid (TBA) was purchased from VWR Chemicals (BDH Chem. Ltd., Poole, UK). Tolbutamide (TBT) was purchased from MedChemExpress (Monmouth Junction, NJ, USA). Additionally, finasteride, dutasteride, minoxidil, and purmorphamine were purchased from Wuhan W&Z Biotech (Wuhan, China).

For cell culture, the primary fibroblast growth medium kit (ready to use) (catalog no. PriMed-iCELL-003) was purchased from iCell Bioscience Inc. (Shanghai, China). Culture media, including Roswell Park Memorial Institute 1640 Medium (RPMI-1640) and Dulbecco’s Modified Eagle Medium (DMEM), along with supplements such as fetal bovine serum (FBS) and penicillin-streptomycin (100×), were acquired from Gibco Life Technologies (Thermo Fisher Scientific, Waltham, MA, USA). The Griess reagent kit for nitric oxide determination was purchased from Invitrogen (Thermo Fisher Scientific, Inc., Eugene, OR, USA). All other reagents employed in the experiments were of analytical purity.

The software used in this study included LabSolutions (version 1.86; Shimadzu, Kyoto, Japan), Image J (version 1.53t; NIH, Bethesda, MD, USA), and Image Lab™ (version 5.1; Bio-Rad Laboratories, Hercules, CA, USA).

### 2.2. Extract Preparation

Perilla seeds, obtained from a local market in Chiang Mai (from November to December 2024), were deposited at the Pharmaceutical and Natural Products Research and Development Unit (PNPRDU), Chiang Mai University (voucher no. PNPRDU670001). The perilla seeds were cleaned with tap water and soaked in aqueous sodium selenite solution at 0 and 80 ppm for 7 days to induce germination, with daily washing using tap water. Germinated perilla seeds treated with selenium at concentrations of 0 and 80 ppm were compared to non-germinated perilla seeds (NG-PS), which were not induced with selenium. After germination, the perilla seeds were dried in an oven at 60 °C for 6 h under controlled humidity to maintain consistent moisture loss.

For SC compression [18], perilla seeds were processed using a screw press machine (LT-RG312, Wenzhou Longqiang Machinery Technology Co., Ltd., Wenzhou, China). The conditions included a die size of 2.5 cm, a pressing temperature of 120 °C, a pressing time of 10 min, and a feed rate of 0.01 g/s (0.05 kg/h). For SFE extraction [19], perilla seeds were processed using the SFE system (Applied Separations, Allentown, PA, USA). In brief, 20 g of perilla seeds were mixed with 9.00 mL of ethanol as a co-solvent, and the mixture was placed into the extraction vessel of the SFE machine. The extraction conditions were set as follows: extraction time of 10 min, temperature of 40 °C, and pressure of 416 bar for all extractions. After both SC and SFE extractions, the eluted extracts were filtered through Whatman filter paper no. 1 and evaporated at 40–50 °C using a rotary evaporator until completely dry. Prior to analysis, the dried extracts were weighed and re-dissolved in 10% DMSO.

All samples were categorized based on the germination condition and extraction method. The experimental groups included non-germinated perilla seeds (NG-PS), germinated perilla seeds in distilled water (0 ppm selenium; G0-PS), and germinated perilla seeds treated with 80 ppm selenium (G80-PS). Each group was extracted using two different techniques: supercritical fluid extraction (SFE) and screw compression (SC), resulting in six sample codes: SFE-NG-PS, SFE-G0-PS, SFE-G80-PS, SC-NG-PS, SC-G0-PS, and SC-G80-PS.

### 2.3. Phytochemical Evaluation

#### 2.3.1. Evaluation of Total Phenolic Content

The total phenolic content in the perilla seed extracts was determined using a modified method adapted from Muangsanguan et al. [2]. Perilla seed extracts were mixed with 10% (*v*/*v*) Folin-Ciocalteu reagent and 6% *(w/v)* saturated sodium bicarbonate and incubated for 2 h. Absorbance was then recorded at 765 nm. Gallic acid standards (10–200 mg/mL) were used to generate a calibration curve, and total phenolic content was expressed as mg gallic acid equivalents per gram of extract.

#### 2.3.2. Evaluation of Tocopherol Composition

The levels of tocopherols were evaluated following a previously described method with minor modifications. The analysis was performed using a Shimadzu HPLC system (Kyoto, Japan) equipped with a fluorescence detector (RF-20A; Shimadzu, Kyoto, Japan). The analysis of tocopherol was carried out using a reversed-phase Ultra C18 column (5 μm, 250 mm × 4.6 mm, Restek, Bellefonte PA, USA). A gradient elution system was applied, employing two solvent mixtures: solvent A (acetonitrile: methanol: isopropanol, 50:40:10) and solvent B (30:65:5). The gradient began with 85% A (15 min), dropped to 10% within 2 min, rose to 50% over 5 min, and returned to 85% within 3 min. The flow rate was set at 1 mL/min, and the total analysis time was 26 min. Detection was performed by fluorescence spectroscopy with excitation and emission wavelengths of 290 nm and 330 nm, respectively [11].

#### 2.3.3. Evaluation of Antioxidant Activities

##### ABTS Radical Scavenging Assay

The ABTS assay was performed according to a previously described method with slight modifications [20]. Briefly, the ABTS stock solution was prepared by mixing equal volumes of 7.0 mM ABTS and 2.45 mM potassium persulfate. The mixture was then stored in the dark at 25 °C for 12–16 h to allow the reaction to complete. The ABTS working solution was adjusted to an absorbance of 0.70 ± 0.02 at 734 nm and freshly prepared before use. Perilla seed extracts were mixed with the solution in a 96-well plate and incubated at 25 °C for 30 min in the dark. Absorbance was measured at 734 nm. Trolox was used as the standard antioxidant, and 95% (*v*/*v*) ethanol served as the blank control. The ABTS radical scavenging activity was calculated according to Equation (1).

(1) $$\mathrm{Scavenging}\;\mathrm{activity}\;(\%)=\frac{\left({\mathrm{Optical}\;\mathrm{density}}_{\;\mathrm{control}}-{\mathrm{Optical}\;\mathrm{density}\;}_{\mathrm{sample}}\right)}{{\mathrm{Optical}\;\mathrm{density}\;}_{\mathrm{control}}}\;\times \;100$$

where ${\mathrm{Optical}\;\mathrm{density}\;}_{\mathrm{control}}$ refers to the absorbance of the radical solution (ABTS or DPPH) mixed with ethanol; ${\mathrm{Optical}\;\mathrm{density}\;}_{\mathrm{sample}}$ refers to the absorbance of the radical solution (ABTS or DPPH) mixed with the perilla seed extract.

##### DPPH Radical Scavenging Assay

The DPPH radical scavenging assay was carried out following a previously described method with minor modifications [20]. The extract was mixed with 0.1 mM DPPH and incubated at 25 °C for 30 min in the dark. Absorbance was measured at 515 nm. Trolox was used as the standard, and ethanol served as the blank. Radical scavenging activity was calculated as described in Equation (1).

#### 2.3.4. Evaluation of Fatty Acid Composition

The fatty acid composition was analyzed according to the method described by Sringarm et al., with slight modifications. Fatty acid methyl esters (FAMEs) were analyzed using a Shimadzu GC-2030 gas chromatograph (Kyoto, Japan) equipped with a flame ionization detector (FID) and a capillary column (RT-2560; 100 m × 0.25 mm × 0.25 µm, Restek, Bellefonte, PA, USA). Helium was used as the carrier gas. The injector and detector temperatures were both set at 250 °C. The oven temperature was initially set at 100 °C and then increased at a rate of 3 °C/min until it reached 240 °C, which was maintained for 20 min. The prepared FAME samples were injected into the chromatograph under these conditions. Chromatographic data were processed using LabSolutions software (version 1.86; Shimadzu, Kyoto, Japan). Individual fatty acid peaks were identified by comparing their retention times with those of known standard mixtures. The results were expressed as the percentage of each fatty acid relative to the total fatty acids present in the sample [21].

### 2.4. In Vitro Cell Viability and Proliferation Assay

Human fibroblast (hTERT), murine macrophage (RAW 264.7), and human prostate cancer cells (DU-145) were obtained from the American Type Culture Collection (Rockville, MD, USA). DU-145 cells were cultured in RPMI-1640, while human fibroblast and RAW 264.7 cells were maintained in DMEM; both media were supplemented with 10% FBS and 1% penicillin/streptomycin (100×). Additionally, primary human hair follicle dermal papilla cells (HFDPCs) were obtained from iCell Bioscience Inc. (Shanghai, China) and cultured in a primary fibroblast growth medium kit.

Cell viability and proliferation were evaluated using the SRB assay following treatment with perilla seed extracts or standard controls at concentrations ranging from 0.063 to 2.000 mg/mL. Cells were plated into 96-well plates (1 × 10^5^ cells/mL) and allowed to adhere. After incubation, the cells were treated with the perilla seed extracts and standard controls for an additional 24 h. Subsequently, cells were fixed with 50% (*w/v*) TCA at 4 °C for 1 h, followed by staining with 0.04% (*w/v*) SRB solution for 30 min. The unbound dye was then removed using 10 mM Tris base. The absorbance was measured at 515 nm. Only concentrations yielding over 80% cell viability were selected for further experiments [11]. The percentage of viable cells was calculated using the following Equation (2).

(2) $$\mathrm{Cell}\;\mathrm{viability}\;\left(\%\right)=\frac{\left({\mathrm{Optical}\;\mathrm{density}}_{\;\mathrm{sample}}-{\mathrm{Optical}\;\mathrm{density}\;}_{\mathrm{blank}}\right)}{\left({\mathrm{Optical}\;\mathrm{density}}_{\;\mathrm{control}}-{\mathrm{Optical}\;\mathrm{density}\;}_{\mathrm{blank}}\right)}\;\times \;100\;$$

### 2.5. Cell Migration Assay

The migration ability of HFDPCs was assessed using a modified scratch assay, as described in previous studies [20]. Briefly, HFDPCs were seeded at a density of 1 × 10^5^ cells/mL into a 24-well plate and incubated for 24 h. After incubation, the cells were washed with 1X PBS (pH 7.2–7.4). A scratch was created in the cell monolayer using a pipette tip, and the plate was rinsed three times with PBS to remove cell debris. Subsequently, perilla seed extracts or the reference compound minoxidil were added to the wells. Cell migration was monitored at 0, 24, and 48 h post-treatment using an inverted microscope. Images were captured and analyzed using Image J software version 1.53t (NIH, Bethesda, MD, USA). The migration rate was calculated using Equation (3).

(3) $$\mathrm{Migration}\;\left(\%\right)=\frac{\left({\mathrm{Area}}_{\;\mathrm{initial}}-{\mathrm{Area}\;}_{\mathrm{at}\;\mathrm{specified}\;\mathrm{time}}\right)}{\left({\mathrm{Area}}_{\;\mathrm{initial}}\right)}\;\times \;100\;$$

### 2.6. TBT-Induced Potassium Channel Blockade Model

The effect of potassium ion channel activation on the viability of HFDPCs was evaluated using a modified potassium ion channel assay [20]. Briefly, HFDPCs were seeded at a density of 1 × 10^5^ cells/mL in a 96-well plate and incubated for 24 h. Cells were pre-treated with 2.5 mM TBT, a potassium channel blocker, for 2 h, followed by exposure to perilla seed extracts or minoxidil for 24 h. After treatment, the SRB cell viability assay protocol was followed. The percentage of viable cells was calculated using Equation (2).

### 2.7. Anti-Inflammatory Activity Assay

A colorimetric assay based on the Griess reaction was used to quantify nitrite accumulation in the supernatant. Macrophage and hair follicle cells were seeded at a density of 1 × 10^5^ cells/mL and incubated for 24 h. Cells were pre-treated with plant extracts, a standard diclofenac sodium, or medium (blank) for 2 h. Subsequently, the cells were stimulated with LPS at a concentration of 1 µg/mL for an additional 24 h. Culture supernatants were harvested. Nitrite levels were measured using the Griess reagent. A calibration curve was prepared from standard nitrite (0.01 to 50 µM) [2].

### 2.8. Thiobarbituric Acid-Reactive Substances (TBARS) Assay

The TBARS assay was used to evaluate the antioxidant potential of perilla seed extracts, using L-ascorbic acid as a standard control and an incomplete medium as the control. HFDPCs were seeded in 6-well plates (1 × 10^5^ cells/mL) and incubated for 24 h. Cells were then treated with 0.125 mg/mL perilla seed extracts or control for 24 h, followed by exposure to H_2_O_2_ for 2 h. Cells were lysed and reacted with a mixture of 1% Triton X-100, TBA, and TCA at 100 °C for 10 min. The reaction was terminated by rapid cooling at −80 °C for 10 min. The absorbance at 532 nm was measured to quantify malondialdehyde (MDA) levels, a key product of lipid peroxidation, relative to the untreated control [2].

### 2.9. Assessment of Gene Expression

Gene expression analysis targeting hair loss-related genes was conducted using the primer sequences listed in Table 1. Briefly, cells were seeded into 6-well plates at a density of 1 × 10^5^ cells/mL and incubated for 24 h. After incubation, the cells were treated with perilla seed extracts or standard controls, including dutasteride, finasteride, minoxidil, and purmorphamine (a standard hair loss treatment), at a concentration of 0.125 mg/mL for an additional 24 h. Total RNA was extracted using the E.Z.N.A.^®^ Total RNA Kit I (Omega BioTek, Norcross, GA, USA), and RNA concentrations were quantified using a NanoDrop Spectrophotometer (Thermo Fisher Scientific, Waltham, MA, USA). Gene expression was subsequently analyzed using semi-quantitative reverse transcription polymerase chain reaction (RT-PCR). cDNA synthesis and PCR amplification were performed using the MyTaq™ One-Step RT-PCR Kit (Bioline, Memphis, TN, USA). PCR products were separated by gel electrophoresis, and band intensities were quantified using the Gel Doc™ EZ System (v3.0; Bio-Rad Laboratories, Hercules, CA, USA) and Image Lab™ software (v5.1). Expression levels were normalized to *GAPDH* and compared against vehicle-treated controls. Results were expressed as fold changes relative to the control group.

### 2.10. Statistical Analysis

All results are expressed as mean ± standard deviation. Statistical comparisons were performed using one-way analysis of variance (ANOVA), followed by Tukey’s HSD test, using SPSS software version 23.0 (Chicago, IL, USA). A *p*-value of less than 0.05 was considered statistically significant.

## 3. Results and Discussion

### 3.1. Extraction Yield, Phytochemical Profile, and Antioxidant Properties of Perilla Seed Extracts

The extraction yields of perilla seed extracts were presented in Table 2. Among the extracts obtained by supercritical fluid extraction (SFE), SFE-G0-PS exhibited the highest extraction yield, followed by SFE-G80-PS and SFE-NG-PS. The results indicated that SFE-derived perilla seed extracts exhibited significantly higher yields compared to those obtained using the screw compression (SC) method. When comparing the effects of selenium-induced germination on extraction yield, SC-G0-PS achieved the highest extraction yield, followed by SC-G80-PS and SC-NG-PS.

The total phenolic content of the perilla seed extracts was also illustrated in Table 2. In this study, SFE-G80-PS exhibited the highest total phenolic content, followed by SFE-NG-PS and SFE-G0-PS. Additionally, extracts obtained from SFE exhibited significantly higher total phenolic content compared to those obtained by SC. For caffeic acid, it was predominantly found in the SFE-PS group, although it was also detected at comparable levels in the SC-G80-PS sample. The superior efficiency of SFE in extracting polyphenols from perilla seed extracts was attributed to several factors, including the use of co-solvents and high pressure, which enhanced polyphenol solubility and yield. Previous studies have also shown that the polarity of co-solvents, such as ethanol, plays a significant role in polyphenol extraction [22]. Among the polyphenolic compounds identified in the SFE, caffeic acid was one of the major constituents. Caffeic acid, a semi-polar compound and one of the polyphenols, has been demonstrated to possess anti-hair loss properties through the androgen pathway by downregulating genes involved in this pathway [23]. Therefore, the high content of caffeic acid in the extract may contribute to its antioxidant properties and potential efficacy against alopecia. However, a comparison of all tested groups (NG-PS, G0-PS, and G80-PS) revealed no statistically significant differences in caffeic acid content. This suggested that selenium treatment, at the concentrations used in this study, did not significantly influence the biosynthesis of caffeic acid or other polyphenols in perilla seeds. Previous studies have reported variable effects of selenium on polyphenol production, with outcomes depending on both selenium concentration and plant species [24].

The amphiphilic nature of vitamin E compounds arises from the presence of hydroxyl groups attached to the aromatic ring and methyl groups on the chromanol ring. The different isoforms of tocopherol, including α-, β-, γ-, and δ-tocopherol, are determined by the number and position of these methyl groups [25]. Among the tocopherol compounds detected in perilla seed extracts, β- and γ-tocopherol, α-tocopherol, and δ-tocopherol were the most abundant, respectively (Table 2). Overall, perilla seed extracts obtained using SFE exhibited significantly higher total tocopherol content compared to those obtained through SC. This result was attributed to the superior efficiency of SFE in extracting non-polar or hydrophobic compounds, such as tocopherols. Supercritical carbon dioxide (SC-CO_2_), the primary solvent used in SFE, is particularly effective in isolating non-polar molecules, including neutral lipids, essential oils, and tocopherols [26]. In contrast, polar compounds, such as phenolics, are less efficiently extracted by this technique [26]. Therefore, the non-polar nature of tocopherols made SFE a more suitable method for their extraction compared to conventional screw extraction. In a comparison among the samples (NG-PS, G0-PS, and G80-PS), no statistically significant differences in total tocopherol content were observed.

Notably, previous studies have demonstrated that tocopherols exhibited strong antioxidant activity [27]. This finding was consistent with our results, as SFE-PS extracts, which had higher tocopherol content, also showed significantly greater antioxidant activity than SC-PS extracts, as measured by both ABTS and DPPH assays. Among the tested perilla seed extracts, the SFE-G80-PS sample exhibited the highest antioxidant activities against ABTS and DPPH radicals, with values of 42.02 ± 0.48% and 56.11 ± 0.18%, respectively. This observation aligned with earlier reports indicating that antioxidant capacity measured by the ABTS assay was often higher than that obtained from the DPPH assay. This difference was attributed to the aqueous nature of the ABTS assay, which more effectively reflected the activity of hydrophilic antioxidants compared to the DPPH assay [28]. The radical scavenging activities observed were closely related to the total phenolic content of each extract. Phenolic acids possess a carboxylic acid group and an aromatic ring with hydroxyl groups, which allow them to stabilize free radicals through electron delocalization—a key mechanism underlying their antioxidant potential [29]. Therefore, the superior antioxidant capacity of the SFE-G80-PS sample corresponded with its higher total phenolic content.

Elevated antioxidant levels have been shown to play a critical role in reducing oxidative stress associated with AGA, potentially delaying premature aging of hair follicles. In HFDPCs, excessive production of reactive oxygen species (ROS) was reported to overwhelm the cells’ intrinsic antioxidant defenses, leading to senescence and impaired follicular activity [30,31]. The strong antioxidant activity and abundance of bioactive compounds in the perilla seed extracts examined in this study highlighted their potential for supporting hair follicle health. Antioxidant-rich plant extracts have been found to reduce oxidative damage and preserve the functional capacity of hair follicle cells, thereby promoting hair regeneration [30]. Regulation of critical molecular pathways related to hair growth, such as Wnt/β-catenin, Sonic Hedgehog, and angiogenesis, has been attributed to these bioactive compounds. Activation of these pathways was found to prolong the anagen phase and improve blood supply to the follicular region, contributing to enhanced hair growth [20].

Polyunsaturated fatty acids were the most abundant, with α-linolenic acid and linoleic acid accounting for approximately 59.44 ± 0.29% and 17.21 ± 0.26%, respectively. Oleic acid (approximately 11.76 ± 0.03%) was the predominant monounsaturated fatty acid, while palmitic acid (approximately 6.94 ± 0.10%) was the most prevalent saturated fatty acid, as shown in Table 3.

Regarding the extraction methods, the fatty acid content of perilla seed extracts obtained from both SPE and SC were similar. However, SFE-PS extracts showed slightly higher values than those obtained through screw extraction. In all tested groups (NG-PS, G0-PS, and G80-PS), the fatty acid content across all tested samples was comparable, with no statistically significant differences observed.

The fatty acid profile observed in this study was consistent with previous reports indicating that α-linolenic acid, linoleic acid, oleic acid, and palmitic acid were the predominant fatty acids in perilla seeds [32]. However, variations in fatty acid content have been attributed to several factors, including cultivar type, geographical origin, climate conditions, and post-harvest processing [33]. Our previous study also showed that SFE yielded slightly higher amounts of fatty acids compared to screw extraction. In contrast, our previous report demonstrated that maceration with ethanol was ineffective in extracting fatty acids from perilla seeds. Moreover, the presence of key fatty acids in this study, such as α-linolenic acid, oleic acid, and palmitic acid, may have contributed to reducing oxidative stress, which is associated with hair loss. Notably, Khantham et al. reported that rice bran extracts rich in fatty acids were able to suppress the expression of *SRD5A*, a gene recognized as a key contributor to the development of androgenetic alopecia [34].

### 3.2. Effect of Perilla Seed Extracts on Cell Viability and Proliferation

Cytotoxicity of perilla seed extracts was assessed through cell viability and proliferation assays across a concentration range of 0.063 to 2.000 mg/mL in RAW 264.7, hTERT-immortalized, DU-145, and HFDPC cells. According to ISO 10993-5 guidelines [35], a cell viability rate above 80% is considered indicative of non-cytotoxicity. After 24 h of treatment, perilla seed extracts at concentrations of ≥0.250 mg/mL exhibited cytotoxic effects in RAW 264.7 and DU-145 cells, whereas concentrations of ≥0.500 mg/mL were cytotoxic to hTERT-immortalized and HFDPC cells, compared to the untreated group. To ensure viability remained above 80% across all four cell types, a concentration of 0.125 mg/mL was selected as the highest non-toxic concentration for subsequent experiments. Detailed HFDPC cell viability data for all concentrations are provided in Supplementary Table S1.

**Figure 1:**
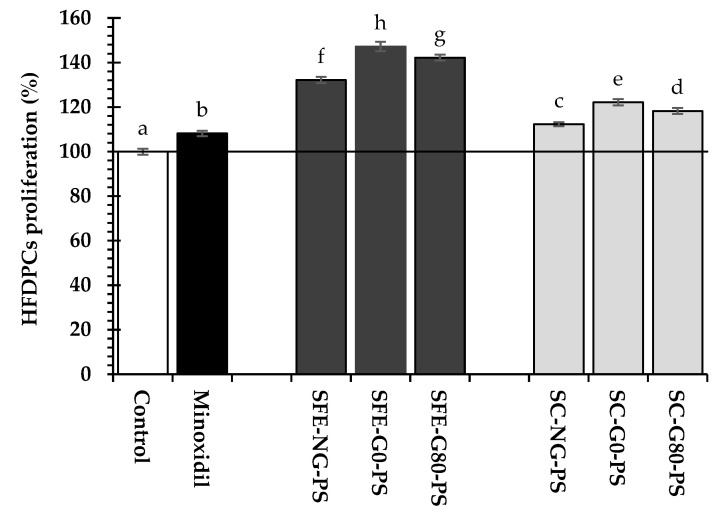
Comparison of HFDPC proliferation in response to perilla seed extracts and the standard treatment (minoxidil) at a concentration of 0.031 mg/mL, relative to the untreated control. Results were expressed as mean ± SD for each sample. One-way ANOVA followed by Tukey’s HSD test was used for statistical analysis. Different letters (a–h) indicate statistically significant differences among samples (*p* < 0.05). SFE-NG-PS: Non-germinated perilla seed extract obtained by supercritical fluid extraction; SFE-G0-PS: Germinated perilla seed extract in distilled water (0 ppm selenium) obtained by supercritical fluid extraction; SFE-G80-PS: Germinated perilla seed extract treated with 80 ppm selenium obtained by supercritical fluid extraction; SC-NG-PS: Non-germinated perilla seed extract obtained by screw compression; SC-G0-PS: Germinated perilla seed extract in distilled water (0 ppm selenium) obtained by screw compression; SC-G80-PS: Germinated perilla seed extract treated with 80 ppm selenium obtained by screw compression.

In addition, all perilla seed extracts significantly stimulated HFDPC proliferation at a concentration of 0.031 mg/mL compared to minoxidil and the untreated control (*p* < 0.05) (Figure 1). Minoxidil is well known to promote HFDPC proliferation by activating the ERK and AKT signaling pathways, protecting cells from apoptosis, and enhancing overall cell growth. HFDPCs are critically involved in hair follicle development, which occurs in three main stages: induction, organogenesis, and cytodifferentiation. During the induction stage, HFDPCs initiate signaling that stimulates the proliferation and differentiation of epithelial cells, leading to the formation of the hair follicle basal plate. In the organogenesis stage, the basal plate expands downward into the dermis to form hair buds, while HFDPCs stimulate keratinocyte proliferation, resulting in the development of dermal papillae. In the final cytodifferentiation stage, the hair follicle fully develops, and the hair shaft emerges through the epidermis, completing the hair follicle formation process.

As previously described, HFDPCs play a crucial role in all stages of hair follicle formation. Among the perilla seed extracts, those obtained via SFE exhibited a greater ability to promote HFDPC proliferation, with rates ranging from 132.16 ± 1.45% to 147.21 ± 2.11%, compared to those from SC, which ranged from 112.32 ± 0.84% to 122.15 ± 1.36%. This enhanced effect may be associated with the presence of bioactive compounds in SFE-PS extracts, including caffeic acid, tocopherols, and fatty acids (particularly linoleic acid and oleic acid). These findings aligned with previous studies suggesting that polyphenols, tocopherols, and fatty acids contribute to HFDPC proliferation. Interestingly, G0-PS exhibited significantly higher proliferation of HFDPCs compared to both G80-PS and NG-PS.

### 3.3. Effect of Perilla Seed Extracts on Cell Migration

The migration of HFDPCs is essential for hair follicle regeneration, particularly during the transition from the telogen (resting) phase to the anagen (growth) phase. During this process, HFDPCs relocate to re-establish contact with the base of the hair follicle, a step that is critical for initiating new hair growth. This migration enables HFDPCs to interact with surrounding epithelial cells, secrete growth factors, and activate signaling pathways necessary for follicular activation, cell proliferation, and vascularization—ultimately supporting robust hair regeneration [36].

**Figure 2:**
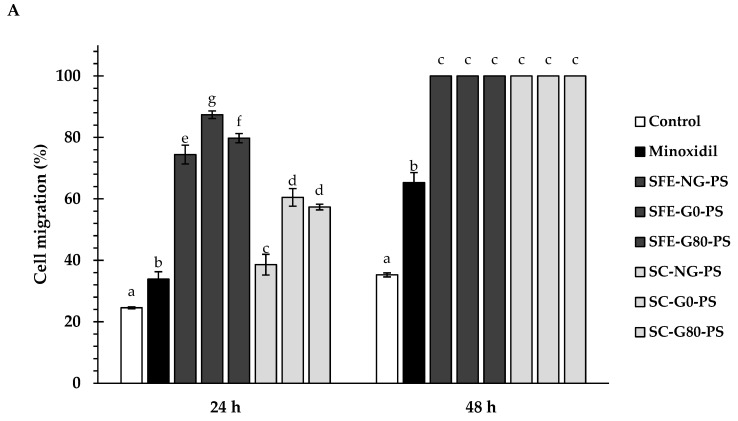

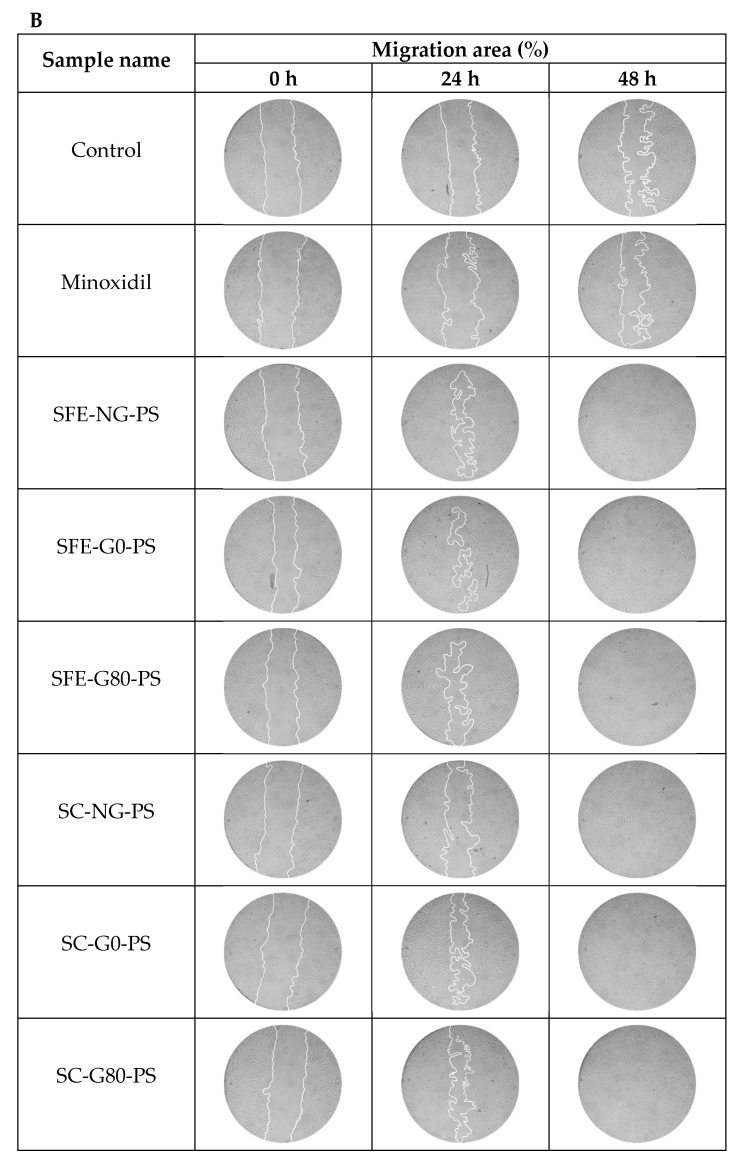
Comparison of HFDPC migration in response to perilla seed extracts and the standard treatment (minoxidil) at a concentration of 0.125 mg/mL, relative to the untreated control. (**A**) Migration percentages at each time point were calculated based on the initial scratch area at 0 h. (**B**) Microscope images showing HFDPC migration areas at the initial, 24 h, and 48 h after treatment with each sample. Results were expressed as mean ± SD for each sample. One-way ANOVA followed by Tukey’s HSD test was used for statistical analysis. Different letters (a–g) indicate statistically significant differences among samples within each treatment time (24 h and 48 h) (*p* < 0.05). SFE-NG-PS: Non-germinated perilla seed extract obtained by supercritical fluid extraction; SFE-G0-PS: Germinated perilla seed extract in distilled water (0 ppm selenium) obtained by supercritical fluid extraction; SFE-G80-PS: Germinated perilla seed extract treated with 80 ppm selenium obtained by supercritical fluid extraction; SC-NG-PS: Non-germinated perilla seed extract obtained by screw compression; SC-G0-PS: Germinated perilla seed extract in distilled water (0 ppm selenium) obtained by screw compression; SC-G80-PS: Germinated perilla seed extract treated with 80 ppm selenium obtained by screw compression.

This study investigated the effects of perilla seed extracts and the standard treatment (minoxidil) on the migration of HFDPCs at a concentration of 0.125 mg/mL (Figure 2). After 24 h, cells treated with perilla seed extracts exhibited significantly higher migration rates (38.59 ± 3.37% to 87.40 ± 1.22%) compared to those treated with minoxidil (33.88 ± 2.41%) (*p* < 0.05). Remarkably, after 48 h, all groups treated with perilla seed extracts showed complete migration, with no remaining wound area, significantly outperforming the minoxidil group (65.29 ± 3.44%). In contrast, the untreated control showed only 35.26 ± 0.66% migration. This enhanced migration effect is likely attributed to the presence of caffeic acid, which has been reported in a previous study to enhance HFDPC migration in hair follicles [37].

### 3.4. Effect of Perilla Seed Extracts on Potassium Ion Channel

Potassium ion (K_ATP_) channels contribute significantly to promoting hair growth by facilitating channel opening, which in turn supports the proliferation of HFDPCs. This process is essential for maintaining hair follicle function and regeneration. However, inhibition of K_ATP_ channels disrupts the mitotic phase of the cell cycle, a critical phase required for cell proliferation, ultimately leading to cell death [38].

**Figure 3:**
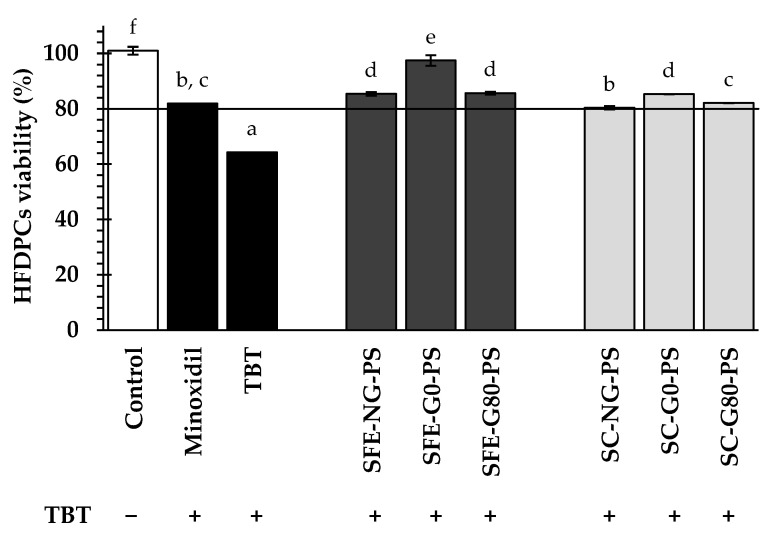
The impact of perilla seed extracts on K_ATP_ channels in HFDPCs was evaluated in comparison to the negative control (2.5 mM TBT alone) and the standard treatment (minoxidil) at a concentration of 0.125 mg/mL. The symbol “**−**” indicated the absence of TBT treatment, while the symbol “+” indicated the presence of TBT treatment. Results were expressed as mean ± SD for each sample. One-way ANOVA followed by Tukey’s HSD test was used for statistical analysis. Different letters (a–f) indicate statistically significant differences among samples (*p* < 0.05). SFE-NG-PS: Non-germinated perilla seed extract obtained by supercritical fluid extraction; SFE-G0-PS: Germinated perilla seed extract in distilled water (0 ppm selenium) obtained by supercritical fluid extraction; SFE-G80-PS: Germinated perilla seed extract treated with 80 ppm selenium obtained by supercritical fluid extraction; SC-NG-PS: Non-germinated perilla seed extract obtained by screw compression; SC-G0-PS: Germinated perilla seed extract in distilled water (0 ppm selenium) obtained by screw compression; SC-G80-PS: Germinated perilla seed extract treated with 80 ppm selenium obtained by screw compression.

In this experiment, TBT, a K_ATP_ channel blocker, was used at a concentration of 2.5 mM to block the channels. TBT has been reported to inhibit hair regeneration, particularly during the anagen phase, by preventing K_ATP_ channel activation. The potential of perilla seed extracts (0.125 mg/mL) to induce K_ATP_ channel opening was evaluated using a cell viability assay based on the SRB method (Figure 3). The results showed that TBT treatment alone (negative control) significantly suppressed HFDPC viability to approximately 64.31 ± 0.01% after 24 h of treatment. In contrast, perilla seed extracts restored HFDPC cell viability after TBT-induced blockade, with values ranging from 80.39 ± 0.06% to 97.49 ± 1.88%. Among the perilla seed extracts, SFE-G0-PS extract exhibited the highest cell viability, followed by SFE-G80-PS and SFE-NG-PS, respectively. Notably, the cell viability observed with SFE-G0-PS was comparable to that of the untreated control (without TBT blockade). This indicates that SFE-G0-PS could potentially counteract the inhibitory effects of TBT and promote HFDPC viability.

Minoxidil, the standard control in this study, is known to activate K_ATP_ channels and influence various tissues. By opening these channels, it promotes the proliferation of HFDPCs, aiding hair follicle development and new hair growth. All perilla seed extracts significantly increased cell viability compared to minoxidil (81.95 ± 0.05%), as shown in Figure 3. As previously described, all perilla seed extracts exerted their effects through a mechanism that appeared more effective than minoxidil. This enhanced effect may be attributed to the synergistic actions of bioactive compounds present in perilla seed extracts, such as caffeic acid and fatty acids, particularly linoleic acid and oleic acid. These findings corresponded to previous research indicating that polyphenols and saturated fatty acids can open K_ATP_ channels, thereby promoting hair regeneration [39,40].

In comparing the extraction methods, the results demonstrated that perilla seed extracts obtained through SFE exhibited significantly higher HFDPC cell viability compared to those obtained via SC. This outcome was likely due to the higher levels of bioactive constituents in SFE-PS extracts, including total phenolic content—especially caffeic acid—along with tocopherols and fatty acids. These findings were in agreement with previous studies suggesting that polyphenols and saturated fatty acids can activate K_ATP_ channels [39,40]. In addition, the highest levels of tocopherols are in the germinated perilla seed extract (G0-PS), which may promote K_ATP_ channel opening. This resulted in a significantly greater increase in HFDPC cell viability for G0-PS compared to G80-PS and NG-PS.

### 3.5. Effect of Perilla Seed Extracts on Anti-Inflammatory Activities

Nitric oxide (NO) has been recognized as a key mediator in hair follicle inflammation and regeneration. Previous research indicated that elevated NO levels in hair follicles were associated with inflammatory responses contributing to hair loss [41]. Oxidative stress, injury, infection, and DHT have been reported to stimulate NO production, thereby promoting inflammation within hair follicles [42]. Under inflammatory conditions, perifollicular macrophages are activated in response to oxidative stress, DHT, or other pro-inflammatory signals. Once activated, these macrophages produce and release significant amounts of NO, which trigger inflammation in the hair follicle, ultimately leading to hair loss [41,42].

**Figure 4:**
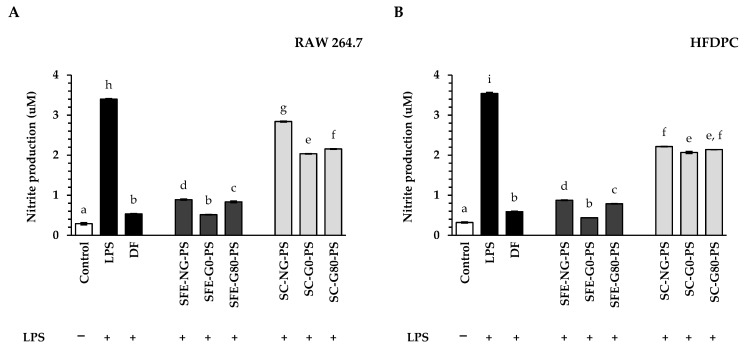
The impact of perilla seed extracts on nitrite suppression in lipopolysaccharide (LPS)-induced (**A**) RAW 264.7 cells and (**B**) HFDPCs was evaluated in comparison to the negative control (LPS-induced without pre-treatment), the standard treatment (DF: diclofenac sodium), and the untreated control at 0.125 mg/mL. The symbol “−” indicated the absence of LPS treatment, while the symbol “+” indicated the presence of LPS treatment. Results were expressed as mean ± SD for each sample. One-way ANOVA followed by Tukey’s HSD test was used for statistical analysis. Different letters (a–i) indicate statistically significant differences among samples (*p* < 0.05). SFE-NG-PS: Non-germinated perilla seed extract obtained by supercritical fluid extraction; SFE-G0-PS: Germinated perilla seed extract in distilled water (0 ppm selenium) obtained by supercritical fluid extraction; SFE-G80-PS: Germinated perilla seed extract treated with 80 ppm selenium obtained by supercritical fluid extraction; SC-NG-PS: Non-germinated perilla seed extract obtained by screw compression; SC-G0-PS: Germinated perilla seed extract in distilled water (0 ppm selenium) obtained by screw compression; SC-G80-PS: Germinated perilla seed extract treated with 80 ppm selenium obtained by screw compression.

In this study, LPS stimulation significantly elevated NO production in both macrophage and hair follicle cells compared to the untreated control (Figure 4A,B). Treatment with perilla seed extracts and the standard anti-inflammatory agent diclofenac sodium (DF) at 0.125 mg/mL effectively suppressed this increase (*p* < 0.05). Among all extracts, SFE-G0-PS demonstrated the greatest inhibitory effect on nitrite production. Interestingly, the nitrite levels in the SFE-G0-PS-treated group were comparable to those observed with DF treatment in both cell types.

In comparing the extraction methods, the results demonstrated that perilla seed extracts obtained through SFE presented a significantly greater reduction in nitrite production compared to those obtained via SC in both RAW 264.7 and HFDPC cells. Previous research has shown that NO levels in hair follicles influence hair regeneration during the anagen phase and contribute to hair loss during the telogen phase [43]. Consistently, SFE-PS, which possessed significantly elevated levels of bioactive constituents, including total phenolic content (notably caffeic acid), also demonstrated enhanced NO suppression. The combination of these bioactive constituents in SFE-PS was previously shown to reduce inflammation by decreasing NO levels in the hair follicles [23,34].

Furthermore, in comparing the different types of perilla seed extracts, the results showed that the G0-PS exhibited the greatest suppression of nitrite levels in both RAW 264.7 and HFDPC cells compared to G80-PS and NG-PS. These findings highlighted the strong potential of G0-PS as a natural anti-inflammatory agent through its ability to suppress nitrite accumulation in cells.

### 3.6. Effect of Perilla Seed Extracts on Antioxidant Activities

Lipid peroxidation is a key process triggered by oxidative stress, in which polyunsaturated fatty acids within the phospholipids of cell membranes react with free radicals, leading to membrane damage. This damage allows free radicals to penetrate cells and accumulate within intracellular structures [44]. To assess lipid peroxidation, the TBARS assay was used to detect MDA, a by-product of lipid peroxidation and a key marker of oxidative stress in cells.

**Figure 5:**
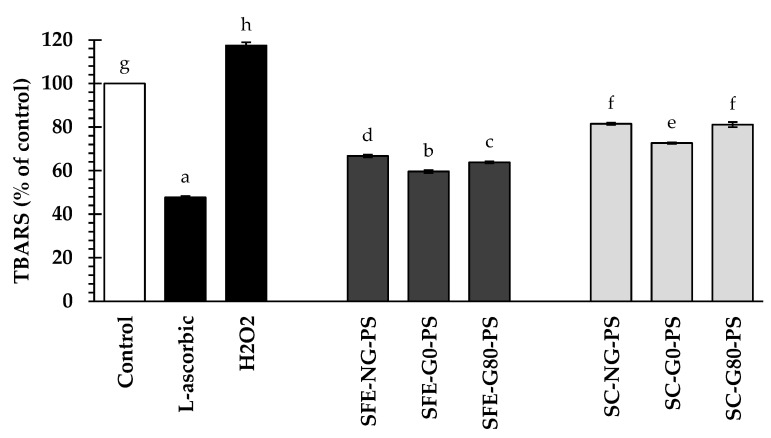
The impact of perilla seed extracts on TBARS suppression in hydrogen peroxide (H_2_O_2_)-induced HFDPCs was evaluated in comparison to the negative control (H_2_O_2_-induced without pre-treatment), standard treatment (L-ascorbic acid), and untreated control, all at a concentration of 0.125 mg/mL. Results were expressed as mean ± SD for each sample. One-way ANOVA followed by Tukey’s HSD test was used for statistical analysis. Different letters (a–h) indicate statistically significant differences among samples (*p* < 0.05). SFE-NG-PS: Non-germinated perilla seed extract obtained by supercritical fluid extraction; SFE-G0-PS: Germinated perilla seed extract in distilled water (0 ppm selenium) obtained by supercritical fluid extraction; SFE-G80-PS: Germinated perilla seed extract treated with 80 ppm selenium obtained by supercritical fluid extraction; SC-NG-PS: Non-germinated perilla seed extract obtained by screw compression; SC-G0-PS: Germinated perilla seed extract in distilled water (0 ppm selenium) obtained by screw compression; SC-G80-PS: Germinated perilla seed extract treated with 80 ppm selenium obtained by screw compression.

To evaluate their antioxidant activity, perilla seed extracts and L-ascorbic acid at 0.125 mg/mL were tested for their ability to suppress MDA formation in H_2_O_2_-treated HFDPCs. As illustrated in Figure 5, the results demonstrated that TBARS levels in the H_2_O_2_-treated group elevated significantly to 117.45 ± 0.50% of the control. Additionally, all perilla seed extracts significantly reduced TBARS levels, ranging from 59.62 ± 0.66 to 81.52 ± 0.51% of the control, indicating their promising potential In oxidative stress reduction. While L-ascorbic acid exhibited the most potent suppression (47.74 ± 0.59% of the control), perilla seed extracts still displayed notable antioxidant activity, highlighting their promise as natural alternatives. Overall, SFE-G0-PS presented significantly greater TBARS suppression compared to other extracts. This superior effect could be attributed to its higher antioxidant capacity, particularly the elevated levels of tocopherols. Previous studies have reported that both tocopherols and polyphenols can reduce lipid peroxidation by-products, thereby protecting cell membranes and lowering oxidative stress [11,45].

When comparing extraction methods, the results demonstrated that SFE yielded perilla seed extracts with significantly greater TBARS reduction compared to the SC method. SFE-PS extracts exhibited significantly higher levels of bioactive constituents, including total phenolic content, particularly caffeic acid, compared to SC-PS, and also showed greater TBARS suppression. These bioactive constituents, particularly polyphenols, in SFE-PS collectively reduced oxidative stress by lowering TBARS levels in hair follicle cells [46,47].

Although the levels of tocopherols and fatty acids were not significantly different among the groups, G0-PS extracts showed the greatest reduction in TBARS levels compared to both G80-PS and NG-PS groups, suggesting a possible contribution of these compounds to the observed effect. These antioxidant-rich compounds likely contributed to the decreased formation of lipid peroxidation by-products, ultimately reducing oxidative stress in cells. This reduction may help attenuate inflammation and protect hair follicles from oxidative damage, thereby preventing hair loss [48].

### 3.7. Effects of Perilla Seed Extracts on Gene Expression Associated with Hair Loss and Hair Growth

Hair formation involved the regeneration and differentiation of hair follicles composed of HFDPC, fibroblasts, and epithelial cells. Hair growth is governed by a cyclic process controlled by molecular signals [49]. This research focused on the major pathways that influence hair regeneration, specifically the androgen pathway, Wnt/β-catenin signaling, Sonic Hedgehog signaling, and angiogenesis.

Hair loss was primarily driven by the action of androgen hormones, with DHT being a key androgenic steroid synthesized by the 5α-reductase (SRD5A) enzyme. Within hair follicles, SRD5A converts testosterone into DHT. Excessive DHT production in androgen-sensitive areas shortened the anagen phase of the hair follicle cycle, leading to follicular miniaturization and, ultimately, hair loss. Three isoforms of SRD5A—SRD5A1, SRD5A2, and SRD5A3—were identified in hair follicles and played a crucial role in AGA [50].

**Figure 6:**
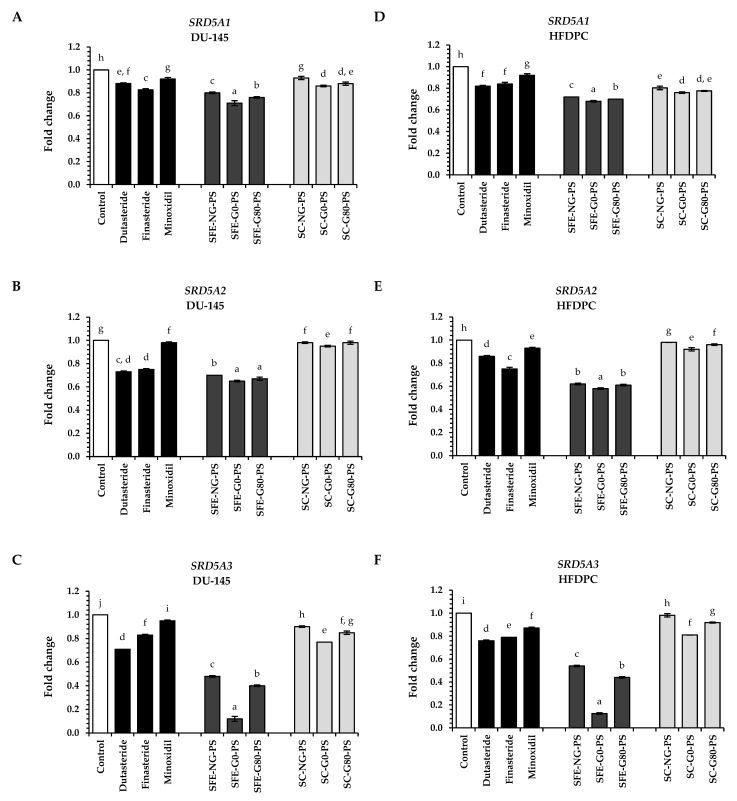
The impact of perilla seed extracts on gene expression in the androgen pathway: (**A**) *SRD5A1*, (**B**) *SRD5A2*, and (**C**) *SRD5A3* in DU-145 cells, and (**D**) *SRD5A1*, (**E**) *SRD5A2*, and (**F**) *SRD5A3* in HFDPCs were evaluated in comparison to standard treatments (dutasteride, finasteride, and minoxidil) at a concentration of 0.125 mg/mL. Results were expressed as mean ± SD for each sample. One-way ANOVA followed by Tukey’s HSD test was used for statistical analysis. Different letters (a–j) indicate statistically significant differences among samples (*p* < 0.05). SFE-NG-PS: Non-germinated perilla seed extract obtained by supercritical fluid extraction; SFE-G0-PS: Germinated perilla seed extract in distilled water (0 ppm selenium) obtained by supercritical fluid extraction; SFE-G80-PS: Germinated perilla seed extract treated with 80 ppm selenium obtained by supercritical fluid extraction; SC-NG-PS: Non-germinated perilla seed extract obtained by screw compression; SC-G0-PS: Germinated perilla seed extract in distilled water (0 ppm selenium) obtained by screw compression; SC-G80-PS: Germinated perilla seed extract treated with 80 ppm selenium obtained by screw compression.

In this study, perilla seed extracts and standard treatments (dutasteride, finasteride, and minoxidil) at a concentration of 0.125 mg/mL were evaluated for their effects on suppressing the expression of *SRD5A1-3* in DU-145 and HFDPC cells. The suppressive effects of perilla seed extracts and standard treatments on androgen pathway genes were presented in Figure 6.

All perilla seed extracts demonstrated a significant reduction in *SRD5A1-3* expression compared to the untreated control (*p* < 0.05), and their suppression of *SRD5A1* was notably greater than observed with standard treatments such as dutasteride, finasteride, and minoxidil (*p* < 0.05). Among all extracts, SFE-G0-PS emerged as the most effective extract in suppressing *SRD5A1-3* gene expression, with fold changes of 0.70 ± 0.02, 0.65 ± 0.01, and 0.13 ± 0.02 in DU-145 cells and 0.68 ± 0.01, 0.57 ± 0.01, and 0.13 ± 0.01 in HFDPC cells, respectively. Notably, the inhibitory activity of SFE-G0-PS was 2.7, 2.8, and 3.2 times greater than that of dutasteride, finasteride, and minoxidil, respectively, across both DU-145 and HFDPC cells, based on the average gene expression levels of *SRD5A1*, *SRD5A2*, and *SRD5A3* (*p* < 0.05). This potent activity may be associated with the presence of high levels of tocopherols and overall fatty acid composition—particularly α-linolenic acid, linoleic acid, oleic acid, and palmitic acid—in the SFE-G0-PS extract, although no statistically significant differences were observed among the bioactive constituents in all extracts. In particular, the *β* + *γ*-tocopherol content (0.56 mg/g extract) was reported to exert strong free radical scavenging activity [48]. These findings corroborated earlier reports indicating that polyphenols, tocopherols, and specific fatty acids can help prevent hair loss by inhibiting the SRD5A enzyme [23,34].

In terms of extraction methods, perilla seed extracts obtained through SFE exhibited significantly stronger suppression of *SRD5A1-3* gene expression and greater biological activity compared to those obtained through the SC method (*p* < 0.05). This observed effect coincided with significantly elevated levels of bioactive compounds in SFE-PS extracts, particularly caffeic acid, tocopherols, and specific fatty acids (*p* < 0.05).

Furthermore, when comparing different types of perilla seed extracts, the results indicated that G0-PS extracts achieved the greatest suppression of *SRD5A1-3* in both cell types compared to the G80-PS and NG-PS groups. Both G0-PS and G80-PS were germinated, a process previously reported to enhance antioxidant accumulation in plants [24]. Our findings revealed that G0-PS contained the highest antioxidant levels, followed by G80-PS and NG-PS. Notably, G0-PS showed the highest concentrations of caffeic acid and *β* + *γ*-tocopherol—compounds previously reported to suppress *SRD5A* expression [23,34].

HFDPCs are essential for the formation and the regeneration of hair follicles. Hair follicle development depends on the interaction among fibroblasts, mesenchymal, and epithelial cells, mediated by signals originating from HFDPCs. A key regulator pathway in this process is the Wnt/β-catenin (CTNNB1) signaling cascade, which regulates the transition of the hair follicles from the resting phase to the growth phase. β-catenin, a central effector in this pathway, is critical for modulating HFDPC metabolism and sustaining follicular activity during the anagen phase. A decrease in β-catenin accumulation in HFDPCs leads to inactivation of Wnt/β-catenin signaling, thereby contributing to hair loss. Conversely, β-catenin in HFDPCs promotes their proliferation and migration, ultimately stimulating hair regeneration [3].

Hair follicle formation is regulated by the Wnt/β-catenin pathway, which acts upstream and triggers the activation of the Sonic Hedgehog (SHH) signaling pathway. This pathway is crucial for mediating communication between mesenchymal and epithelial cells. The Sonic Hedgehog signaling pathway not only promotes hair follicle formation and repair but also helps maintain the stem cell properties of hair follicle bulge cells. In this cascade, SHH ligands bind to the Patched (PTCH) receptor, relieving inhibition of the Smoothened (SMO) protein. Upon activation, SMO dissociates and binds to the Ellis-van Creveld syndrome (EVC) complex before relocating to the primary cilium. This event triggers the activation of glioma-associated oncogene (GLI) transcription factors and protein Kinase A (PKA), resulting in the assembly of a macromolecular complex. Finally, the GLI complex translocates into the nucleus of HFDPCs, initiating the transcription of genes that drive the transition of hair follicles from the resting phase to the growth phase [3].

In addition, vascular endothelial growth factor (VEGF) is a key angiogenic factor that supports hair follicle formation during the growth phase. VEGF stimulates the neovascularization around the follicles, ensuring adequate delivery of oxygen and nutrients to the hair follicle. Furthermore, VEGF also enhances follicular growth, increases hair shaft diameter, and accelerates overall hair growth [51].

**Figure 7:**
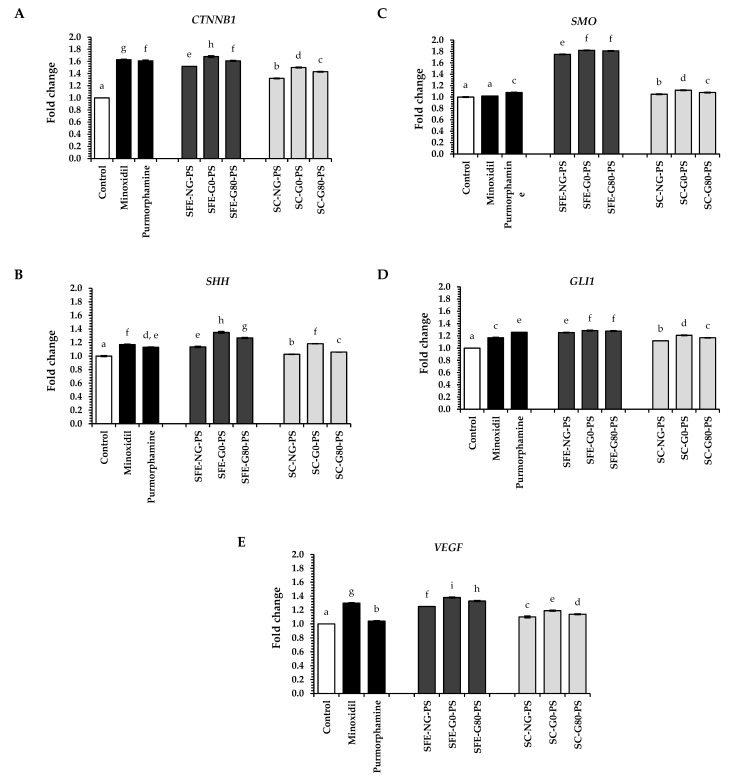
The impact of perilla seed extracts on gene expression in the hair regeneration pathway, including Wnt/β-catenin (**A**) *CTNNB1*; Sonic Hedgehog (**B**) *SHH,* (**C**) *SMO,* (**D**) *GLI1*; and angiogenesis (**E**) *VEGF* in HFDPCs was evaluated in comparison to standard treatments (minoxidil and purmorphamine) at a concentration of 0.125 mg/mL. Results were expressed as mean ± SD for each sample. One-way ANOVA followed by Tukey’s HSD test was used for statistical analysis. Different letters (a–i) indicated statistically significant differences among samples (*p* < 0.05). SFE-NG-PS: Non-germinated perilla seed extract obtained by supercritical fluid extraction; SFE-G0-PS: Germinated perilla seed extract in distilled water (0 ppm selenium) obtained by supercritical fluid extraction; SFE-G80-PS: Germinated perilla seed extract treated with 80 ppm selenium obtained by supercritical fluid extraction; SC-NG-PS: Non-germinated perilla seed extract obtained by screw compression; SC-G0-PS: Germinated perilla seed extract in distilled water (0 ppm selenium) obtained by screw compression; SC-G80-PS: Germinated perilla seed extract treated with 80 ppm selenium obtained by screw compression.

All perilla seed extracts significantly upregulated the expression of genes involved in key hair regeneration pathways, including *CTNNB1* (Wnt/β-catenin), *SHH*, *SMO*, *GLI1* (Sonic Hedgehog), and *VEGF* (angiogenesis), as shown in Figure 7A–E. Statistical significance was observed in all comparisons (*p* < 0.05). Among all extracts, treatment with SFE-G0-PS exhibited the highest expression across all tested genes. The fold changes for *CTNNB1*, *SHH*, *SMO*, *GLI1*, and *VEGF* were 1.68 ± 0.01, 1.35 ± 0.01, 1.82 ± 0.01, 1.29 ± 0.01, and 1.38 ± 0.01, respectively.

Notably, SFE-G0-PS treatment showed approximately 1.0- to 1.3-times greater activity than minoxidil and purmorphamine, standard controls used in this study, in promoting hair regeneration-related pathways (*p* < 0.05). This enhanced activity may be attributed to SPE-G0-PS extract’s rich fatty acid content, particularly α-linolenic acid (59.81%), linoleic acid (17.50%), oleic acid (11.75%), and palmitic acid (6.83%), all of which have been reported to promote the expression of genes involved in hair regeneration. These findings aligned with previous studies indicating the beneficial impact of polyunsaturated and monounsaturated fatty acids on hair follicle stimulation [34].

Regarding the extraction methods, SFE-PS extracts primarily influenced the elevation of gene expression levels in hair regeneration pathways, indicating that SFE is the most efficient method for extracting key compounds beneficial to promoting hair regeneration activity of perilla seed extracts. In comparing the types of perilla seed extracts, G0-PS showed the greatest enhancement in gene expression related to hair regeneration in HFDPCs, followed by G80-PS and NG-PS. These results suggested that the germination process can increase the levels of bioactive molecules associated with hair regeneration pathways. However, despite the lower total phenolic content and antioxidant capacity of G0-PS compared to G80-PS, G0-PS consistently demonstrated stronger biological effects in cell-based assays. This suggests that the presence or balance of specific bioactive compounds, rather than their overall abundance, may play a more critical role in hair growth-related mechanisms. It is possible that certain compounds in G0-PS—such as α-linolenic acid, palmitic acid, or tocopherol isomers—act synergistically or are more bioavailable, thereby contributing to its superior activity. Further investigations focusing on compound-specific effects and bioavailability are warranted to better understand these observations.

## 4. Conclusions

This study demonstrated that *Perilla frutescens* seed extracts are a rich source of bioactive compounds—particularly polyphenols, tocopherols, and fatty acids—with promising biological activities relevant to hair growth promotion. Regarding extraction methods, perilla seed extracts obtained via supercritical fluid extraction (SFE) exhibited higher levels of bioactive compounds and antioxidant capacity compared to those obtained via screw compression (SC). Among the germinated groups, the extract from germinated perilla seeds in distilled water (0 ppm selenium; G0) showed the greatest potential in in vitro assays related to hair loss prevention and hair growth promotion, followed by the germinated with 80 ppm selenium extract (G80) and the non-germinated extract (NG). Notably, the SFE-G0-PS extract demonstrated the strongest potential in multiple in vitro models. These included the enhancement of human dermal papilla cell (HFDPC) proliferation, migration, and survival, particularly under K_ATP_ channel inhibition. Additionally, SFE-G0-PS exerted potent antioxidant and anti-inflammatory effects, and modulated molecular targets associated with hair loss and regeneration. Notably, SFE-G0-PS reduced TBARS and nitrite levels in HFDPCs, indicating protective effects against oxidative stress and inflammation in hair follicles. It also suppressed androgen-related genes (*SRD5A1-3*) in DU-145 and HFDPCs, while upregulating Wnt/β-catenin (*CTNNB1*), Sonic Hedgehog (*SHH*, *SMO*, and *GLI1*), and *VEGF* in HFDPCs, supporting its role in hair regeneration. In summary, our findings highlighted the importance of extraction methods in influencing the phytochemical profile and bioactivities of perilla seed extracts. The germination process further enhanced their therapeutic potential. Among all tested samples, SFE-G0-PS emerged as the most promising candidate for promoting hair growth and preventing hair loss. Further studies should be conducted to elucidate the molecular mechanisms of each bioactive constituent in perilla seed extracts to fully understand the therapeutic potential of SFE-G0-PS as an active ingredient in hair loss treatment and hair growth promotion.

## Figures and Tables

**Table 1:** Primer sequences utilized for semi-quantitative RT-PCR analysis.

Modulated Signaling Pathways	Genes	Accession No.	Forward Sequences	Reverse Sequences
Androgen pathway	*SRD5A1*	NM_001047.4	AGCCATTGTGCAGTGTATGC	AGCCTCCCCTTGGTATTTTG
	*SRD5A2*	NM_000348.4	TGAATACCCTGATGGGTGG	CAAGCCACCTTGTGGAATC
	*SRD5A3*	NM_024592.5	TCCTTCTTTGCCCAAACATC	TCCTTCTTTGCCCAAACATC
Wnt/β-catenin	*CTNNB1*	NM_001330729.2	CCCACTAATGTCCAGCGTTT	AACCAAGCATTTTCACCAGG
Sonic Hedgehog	*SHH*	NM_000193.4	AAAAGCTGACCCCTTTAGCC	GCTCCGGTGTTTTCTTCATC
	*SMO*	NM_005631.5	GAAGTGCCCTTGGTTCGGACA	CCGCCAGTCAGCCACGAAT
	*GLI1*	NM_005269.3	GCAGGGAGTGCAGCCAATACAG	GAGCGGCGGCTGACAGTATA
Angiogenesis	*VEGF*	NM_001025366.3	CTACCTCCACCATGCCAAGT	GCGAGTCTGTGTTTTTGCAG
Internal control	*GAPDH*	NM_001289745.3	GGAAGGTGAAGGTCGGAGTC	CTCAGCCTTGACGGTGCCATG

**Table 2:** Yield, bioactive constituents, and antioxidant activities of perilla seed extracts.

Results		Perilla Seed Extracts					
		SFE-NG-PS	SFE-G0-PS	SFE-G80-PS	SC-NG-PS	SC-G0-PS	SC-G80-PS
Extraction yield (%)		33.93 ± 0.28	40.03 ± 0.39	39.56 ± 0.48	22.46 ± 0.20	25.06 ± 0.14	24.04 ± 0.25
Total phenolic content (mg GAE/g extract)		8.22 ± 0.73 ^d^	6.92 ± 1.38 ^c^	11.31 ± 0.38 ^e^	1.17 ± 0.39 ^a^	2.22 ± 0.36 ^b^	2.64 ± 1.17 ^b^
Caffeic acid (µg/g extract)		12.80 ± 0.03 ^a^	12.39 ± 0.03 ^a^	12.57 ± 0.03 ^a^	ND	ND	11.98 ± 0.03 ^a^
Tocopherol (mg/g extract)	α-Tocopherol	0.02 ± 0.00 ^a^	0.02 ± 0.00 ^a^	0.02 ± 0.00 ^a^	0.03 ± 0.00 ^b^	0.02 ± 0.00 ^a^	0.02 ± 0.00 ^a^
	*β* + *γ*-Tocopherol	0.49 ± 0.00 ^a^	0.56 ± 0.00 ^e^	0.55 ± 0.05 ^d^	0.50 ± 0.00 ^b^	0.53 ± 0.00 ^c^	0.50 ± 0.01 ^b^
	δ-Tocopherol	0.01 ± 0.00 ^a^	0.01 ± 0.00 ^a^	0.01 ± 0.00 ^a^	0.01 ± 0.00 ^a^	0.01 ± 0.00 ^a^	0.01 ± 0.00 ^a^
Antioxidant activities (%)	ABTS	38.30 ± 0.57 ^d^	39.11 ± 3.25 ^e^	42.02 ± 0.48 ^f^	28.89 ± 1.82 ^a^	36.20 ± 0.06 ^c^	35.39 ± 0.86 ^b^
	DPPH	45.29 ± 0.72 ^d^	48.09 ± 0.72 ^e^	56.11 ± 0.18 ^f^	32.19 ± 0.90 ^a^	35.77 ± 1.08 ^c^	35.75 ± 2.34 ^b^

Results were expressed as mean ± SD for each sample. One-way ANOVA followed by Tukey’s HSD test was used for statistical analysis. Different letters (a–f) indicated statistically significant differences among samples within each experiment (*p* < 0.05). mg GAE/g extract: Milligrams of gallic acid equivalents per gram of extract; mg/g extract: Milligrams per gram of extract; SFE-NG-PS: Non-germinated perilla seed extract obtained by supercritical fluid extraction; SFE-G0-PS: Germinated perilla seed extract in distilled water (0 ppm selenium) obtained by supercritical fluid extraction; SFE-G80-PS: Germinated perilla seed extract treated with 80 ppm selenium obtained by supercritical fluid extraction; SC-NG-PS: Non-germinated perilla seed extract obtained by screw compression; SC-G0-PS: Germinated perilla seed extract in distilled water (0 ppm selenium) obtained by screw compression; SC-G80-PS: Germinated perilla seed extract treated with 80 ppm selenium obtained by screw compression.

**Table 3:** Percentage of fatty acid composition in perilla seed extracts.

Classification	Name	SFE-NG-PS	SFE-G0-PS	SFE-G80-PS	SC-NG-PS	SC-G0-PS	SC-G80-PS
Saturated Fatty Acids	Pentadecanoic acid	0.01	0.01	0.01	0.01	0.01	0.01
	Palmitic acid	6.83	7.08	7.03	6.88	6.94	6.89
	Heptadecanoic acid	0.14	0.15	0.15	0.14	0.16	0.15
	Stearic acid	2.88	3.05	3.00	2.90	2.92	2.75
	Arachidic acid	0.18	0.19	0.18	0.18	0.19	0.17
	Behenic acid	0.02	0.02	0.01	0.03	0.01	0.02
	Heneicosanoic acid	0.05	0.06	0.06	0.05	0.06	0.06
	Butyric acid	0.76	0.83	1.20	0.86	0.92	1.07
	Myristic acid	0.02	0.02	0.02	0.02	0.02	0.02
Monounsaturated Fatty Acids	Palmitoleic acid	0.01	0.01	0.02	0.01	0.02	0.01
	Heptadecenoic acid	0.01	0.01	0.01	0.01	0.01	0.01
	Oleic acid	11.75	11.76	11.75	11.74	11.82	11.73
	Gondoic acid	0.04	0.05	0.05	0.07	0.03	0.06
	Erucic acid	0.03	0.00	0.00	0.01	0.01	0.01
	Nervonic acid	0.01	0.01	0.01	0.01	0.01	0.01
Polyunsaturated Fatty Acids	Trans-Linoleic acid	0.07	0.07	0.07	0.06	0.08	0.07
	Linoleic acid	17.45	17.50	16.90	17.36	16.98	17.09
	γ-Linolenic acid	0.21	0.21	0.21	0.21	0.22	0.22
	α-Linolenic acid	59.43	59.81	59.50	59.23	59.75	59.72
	Eicosadienoic acid	0.01	0.11	0.14	0.02	0.11	0.28
	Dihomo-γ-linolenic acid	0.02	0.03	0.03	0.02	0.02	0.02
	Eicosatrienoic acid	0.01	0.00	0.00	0.01	0.00	0.01
	Eicosapentaenoic acid	0.02	0.02	0.01	0.02	0.02	0.01
	Docosahexaenoic acid	0.01	0.00	0.00	0.01	0.01	0.00

The results are expressed as the percentage (%) of total fatty acid content in each sample. SFE-NG-PS: Non-germinated perilla seed extract obtained by supercritical fluid extraction; SFE-G0-PS: Germinated perilla seed extract in distilled water (0 ppm selenium) obtained by supercritical fluid extraction; SFE-G80-PS: Germinated perilla seed extract treated with 80 ppm selenium obtained by supercritical fluid extraction; SC-NG-PS: Non-germinated perilla seed extract obtained by screw compression; SC-G0-PS: Germinated perilla seed extract in distilled water (0 ppm selenium) obtained by screw compression; SC-G80-PS: Germinated perilla seed extract treated with 80 ppm selenium obtained by screw compression.

## Data Availability

Data are contained within this article and the Supplementary Materials. Further inquiries can be directed to the corresponding author.

## Supplementary Materials

The following supporting information can be downloaded at: https://www.mdpi.com/article/10.3390/biology14070889/s1, Table S1: Cell viability responses of HFDPCs to perilla seed extracts at all tested concentrations.
