# Supplementary material for: Germination Enhances Phytochemical Profiles of Perilla Seeds and Promotes Hair Growth via 5α-Reductase Inhibition and Growth Factor Pathways
Source: Biology (Basel). 2025 Jul 20;14(7):889. doi: 10.3390/biology14070889 (PMC12292940; doi:10.3390/biology14070889)
Supplement: Supplementary file 1 [file biology-14-00889-s001.zip › biology-3718530-supplementary.pdf]

Article

Germination Enhances the Phytochemical Profile of Perilla Seeds and Promotes Hair Growth via 5α-Reductase Inhibition and Growth Factor Pathways

Supplementary Information

Table S1. Cell viability responses of HFDCPs to perilla seed extracts at all tested concentrations.

Concentration of perilla seed extracts (mg/mL)	Cell viability (% of control)					
	**SFE-NG-PS**	**SFE-G0-PS**	**SFE-G80-PS**	**SC-NG-PS**	**SC-G0-PS**	**SC-G80-PS**
2.000	39.30 ± 0.79	55.96 ± 0.84	49.27 ± 1.32	0.39 ± 0.09	66.87 ± 1.38	7.07 ± 0.06
1.000	56.23 ± 1.44	76.28 ± 1.19	56.57 ± 1.96	37.62 ± 0.83	100.60 ± 1.95	17.26 ± 0.14
0.500	73.08 ± 1.14	84.88 ± 2.38	78.17 ± 2.00	72.85 ± 0.68	100.70 ± 2.32	34.28 ± 0.12
0.250	104.26 ± 1.64	100.75 ± 2.06	104.54 ± 1.99	81.05 ± 1.34	100.56 ± 2.27	91.72 ± 3.27
0.125	101.51 ± 1.61	110.57 ± 2.03	100.65 ± 2.09	99.74 ± 1.66	98.67 ± 1.68	95.43 ± 0.37
0.063	101.97 ± 1.73	114.55 ± 0.47	96.15 ± 1.49	101.69 ± 2.60	100.12 ± 2.08	98.61 ± 0.23
0.031	121.10 ± 1.80	136.30 ± 1.95	129.58 ± 1.82	117.66 ± 1.54	128.32 ± 1.60	123.76 ± 0.31
0.016	99.45 ± 1.69	104.35 ± 1.91	103.36 ± 2.13	96.97 ± 1.64	97.64 ± 2.13	98.66 ± 0.41
0.008	98.41 ± 1.49	100.06 ± 1.61	98.76 ± 1.52	100.61 ± 1.73	97.16 ± 2.08	100.33 ± 0.20
0.004	95.16 ± 1.42	95.55 ± 1.57	98.71 ± 2.09	100.51 ± 1.94	97.73 ± 1.95	100.57 ± 0.35

Results were expressed as mean ± SD for each sample. ; SFE-NG-PS: Non-germinated perilla seed extract obtained by supercritical fluid extraction; SFE-G0-PS: Germinated perilla seed extract in distilled water (0 ppm selenium) obtained by supercritical fluid extraction; SFE-G80-PS: Germinated perilla seed extract treated with 80 ppm selenium obtained by super-critical fluid extraction; SC-NG-PS: Non-germinated perilla seed extract obtained by screw compression; SC-G0-PS: Germinated perilla seed extract in distilled water (0 ppm selenium) obtained by screw compression; SC-G80-PS: Germinated perilla seed extract treated with 80 ppm selenium obtained by screw compression.
